# Delayed Presentation of Traumatic Intraperitoneal Rupture of Urinary Bladder

**DOI:** 10.1155/2012/430746

**Published:** 2012-12-05

**Authors:** Hazim H. Alhamzawi, Husham M. Abdelrahman, Khalid M. Abdelrahman, Ayman El-Menyar, Hassan Al-Thani, Rifat Latifi

**Affiliations:** ^1^Trauma Surgery Section, Department of Surgery, Hamad Medical Corporation, P.O. Box 3050, Doha, Qatar; ^2^Urology Section, Surgery Department, Hamad Medical Corporation, P.O. Box 3050, Doha, Qatar; ^3^Clinical Research, Trauma Surgery Section, Hamad Medical Corporation, Doha, Qatar; ^4^Clinical Medicine, Weill Cornell Medical School, P.O. Box 24144, Doha, Qatar

## Abstract

Blunt injury of the urinary bladder is well known and usually associates pelvic fractures. Isolated bladder injury is a rare condition and on the other hand, delayed bladder perforation is an extremely rare entity. Herein, we described an unusual case of isolated delayed intraperitoneal bladder rupture that occurred on the third post injury day in a young male in the absence of free intraperitoneal fluid and pelvic fracture. The diagnostic workup, course and the need for surgical repair of the injury is presented.

## 1. Introduction

Around 60% to 85% of all bladder injuries result from blunt abdominal trauma (BAT) but the incidence of intraperitoneal urinary bladder (UB) rupture is relatively uncommon from blunt injuries [1]. Isolated UB rupture following blunt trauma has an insidious presentation, and often results in delayed diagnosis and management [2–8]. The mechanism of injury include sudden compression of the full bladder, shear forces, or a pelvic fracture [2, 3, 9].

Rupture of bladder may be presented with lower abdominal pain, inability to void, and perineal ecchymoses [3]. The cardinal sign of injury to the bladder is gross hematuria [6], which is present in more than 95% of cases, while only about 5% of the patients have microscopic hematuria alone [6, 7]. Over 80% of the patients with UB rupture had an associated pelvic fracture in centers with high percentage of blunt trauma. On the other hand around 6% of patients with pelvic fracture sustain a bladder injury [3, 6]. 

Diagnosis of bladder injury, several days after admission, could be either a missed diagnosis or a truly delayed rupture. Delayed diagnosis of bladder rupture may be associated with laboratory abnormalities such as metabolic derangements, and leukocytosis. Delay in the presentation and treatment may substantially increases mortality [7–10]. Therefore, early and accurate diagnosis with imaging techniques is imperative. Computed tomographic cystography (CTC) and/or retrograde cystography (RGC) are the standard imaging tools for the diagnosis of bladder injury [4–10]. We present a case of delayed rupture of UB due to blunt trauma without associated injuries.

## 2. Case Report

A twenty three-year old male patient sustained BAT due to fall from a 3-meter height. Initial vital signs were: blood pressure136/80 mmHg, heart rate 64 BPM, respiratory rate 20 per minute, oxygen saturation of 100% on room air, and temperature of 36.9°C. Patient was fully conscious with neither external bleeding nor neurological deficits. Abdominal examination showed mild generalized tenderness and voluntary guarding at epigastric and suprapubic regions. Pelvic, genitourinary & rectal examinations revealed no abnormality.

**Figure 1:**
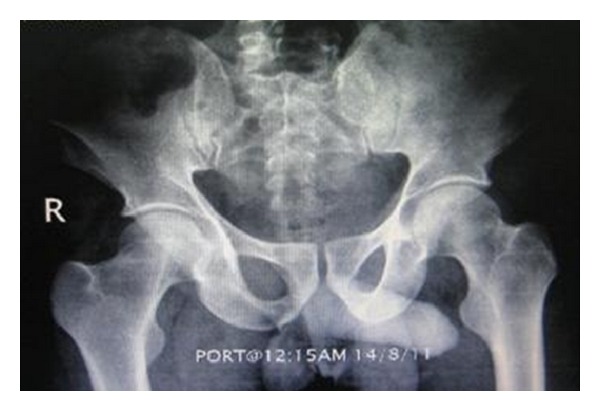
Pelvic X-ray of the patient showing no fracture.

**Figure 2:**
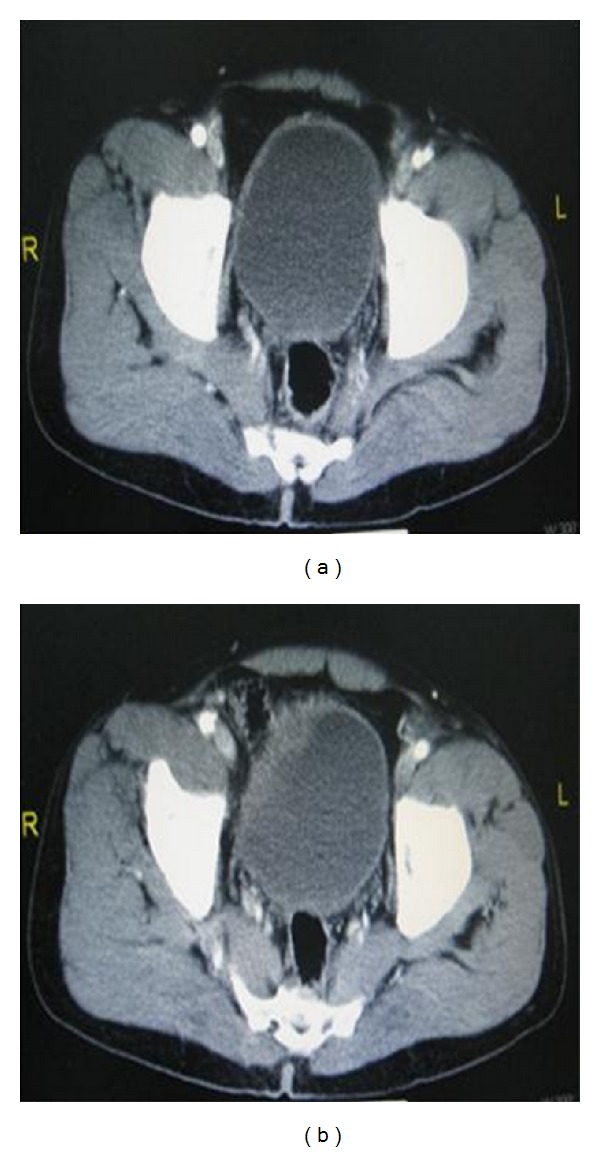
(a) CT image outlines the distended urinary bladder (UB). (b) CT image showing irregularity and thickening on the right side of UB wall.

Initial chest and pelvic X-rays (Figure 1) and focused abdominal ultrasonography for trauma (FAST) were unremarkable. Routine laboratory investigations were unremarkable. CT abdomen and pelvis showed no bony injuries and no intraperitoneal free fluid or air (Figures 2(a) and 2(b)). A Foley's catheter was inserted and revealed frank hematuria that was clearing up. Patient was reassessed 6 hours later; pain was remarkably reduced and he tolerated soft diet with no vomiting, passed motion and had no fever. Abdominal examination revealed mild suprapubic tenderness and normal bowel sound.

**Figure 3:**
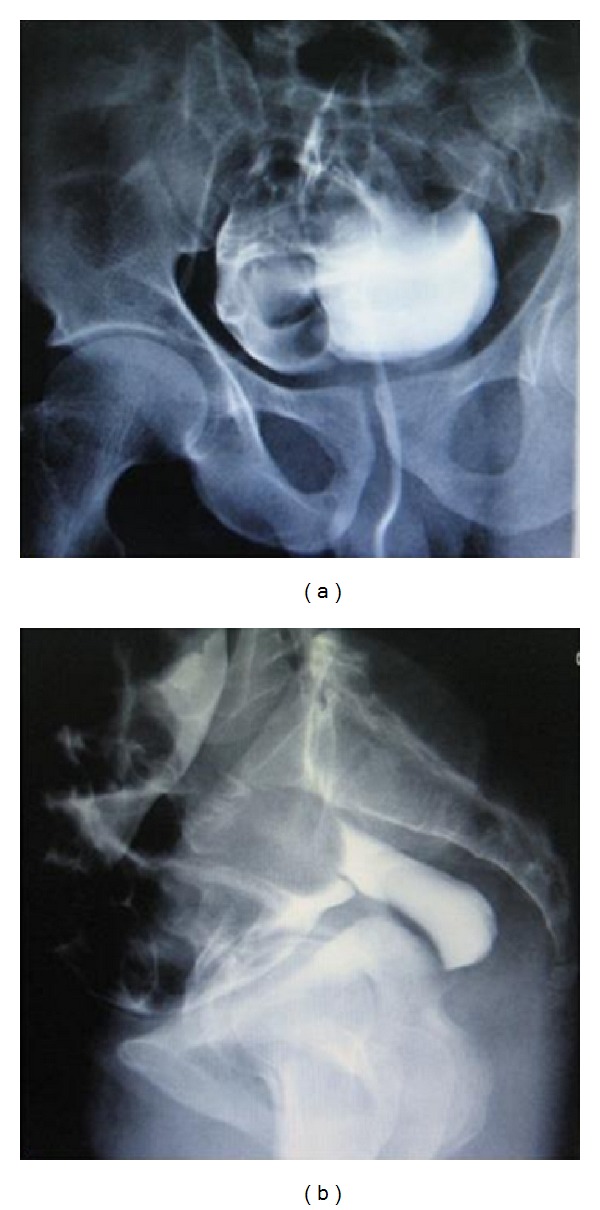
Conventional retrograde cystogram showing extravasation of contrast material outside the UB, denoting intraperitoneal UB rupture.

**Figure 4:**
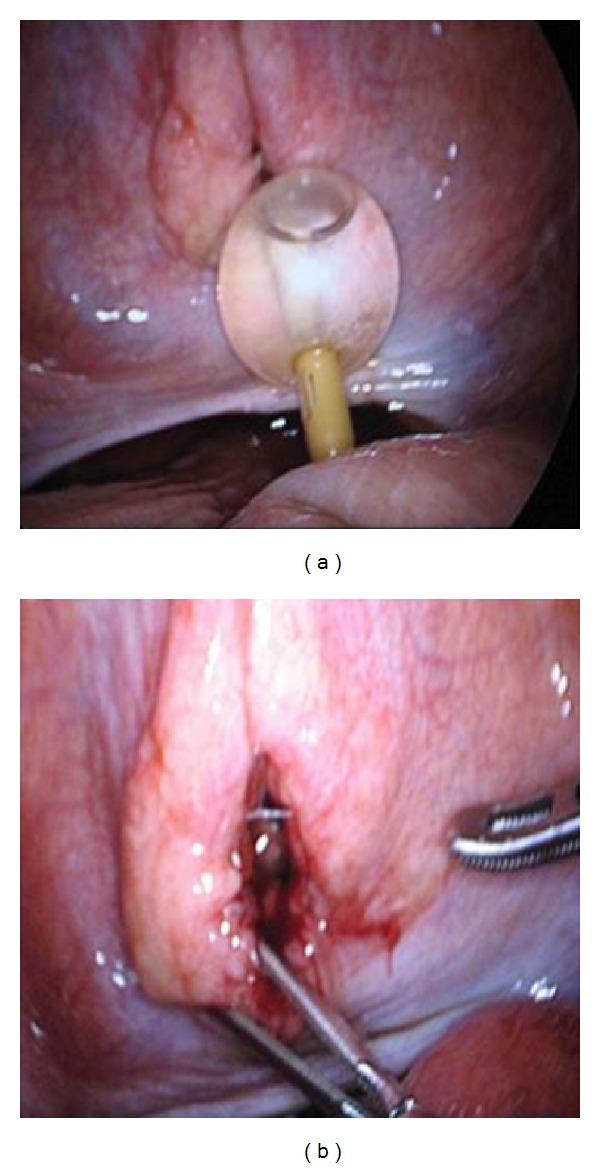
(a) Laparoscopic view showing Foley's catheter balloon outside the UB and intraperitoneally. (b) The tear in the UB wall which was repaired laparoscopically.

**Figure 5:**
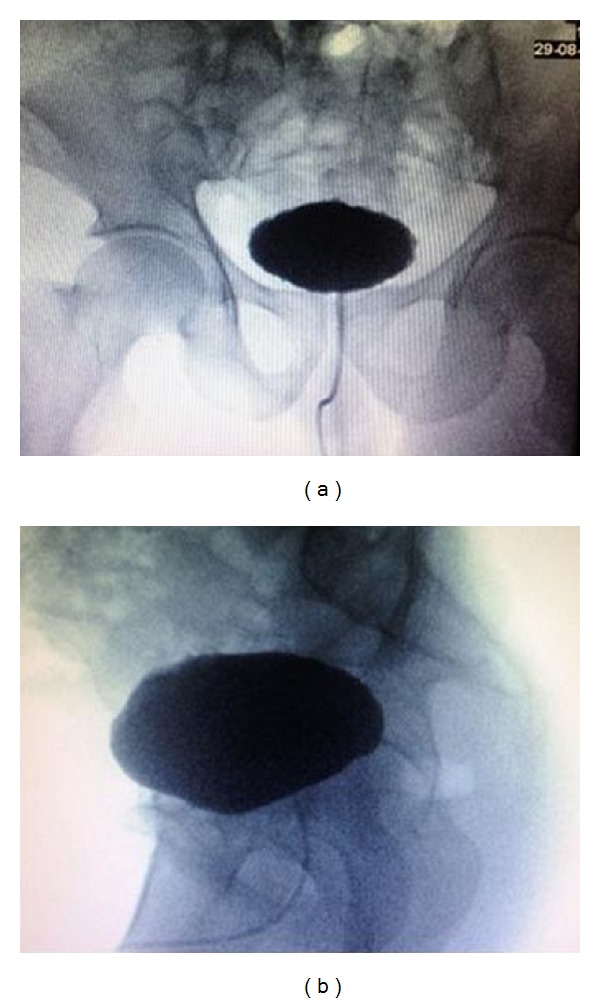
Retrograde cystography showing no contrast extravasation after 10 days of the repair.

On the following day, Foley's catheter was removed for a short duration. Consequently he developed urine retention and hence a Foley catheter was inserted with immediate drainage of 850 mL of clear urine. Repeated CT scan of abdomen reported no free fluid or air within the abdomen. However, the Foley's catheter was found crossing the UB wall. A formal cystogram was done and revealed free intraperitoneal extravasation of contrast denoting intraperitoneal UB perforation (Figures 3(a) and 3(b)). Laparoscopic exploration showed a linear tear of 3 cm at the dome of the UB with the Foley's catheter balloon floating freely in the peritoneum through the tear (Figures 4(a) and 4(b)). Laparoscopic repair was done using 2/0 Vicryl suture in a continuous interlocking water tight fashion and no drains were left. Foley was removed on the tenth post-operative day, a following a normal cystogram (Figures 5(a) and 5(b)). Postoperative follow up was unremarkable.

## 3. Discussion

We present a rare case of delayed intraperitoneal bladder rupture post blunt abdominal trauma. To the best of our knowledge this is the fourth case in the literature [11–14]. Bladder injuries are not uncommon and have different types (I-V) according to the presence of contusion (I), intraperitoneal perforation (II), interstitial injury (III), extraperitoneal rupture (IV) or combined (V) [15].

Differentiation of extraperitoneal from intraperitoneal is essential for the management of bladder injuries. Extraperitoneal rupture is treated conservatively while intraperitoneal rupture needs urgent repair [16]. As CT scan is the standard tool for evaluation of stable blunt abdominal injuries, CT cystogram is considered the test of choice to diagnose bladder injuries [16]. Unfortunately, this modality failed to demonstrate any free intraperitoneal fluid and no dye extravasations in the current case. However, in a retrospective review, subtle focal bladder wall thickening was observed in the admission CT scan (Figure 2(b)). Our hypothesis is that the bladder wall injury in our patient was initially a partial contusion that had progressed after urine retention into a full thickness perforation (LaPlace law) on the third day after admission.

Our case is unique in the absence of associated pelvic fracture in comparison to the previously reported cases [11, 12]. 

## 4. Conclusion

Although rare, delayed bladder injury presentation is possible and one should have high index of suspicion in trauma patient with unexplained abdominal findings and or urinary retention.
